# Optimization of vertically aligned carbon nanotube beam trajectory with the help of focusing electrode in the microchannel plate

**DOI:** 10.1038/s41598-023-42554-8

**Published:** 2023-09-20

**Authors:** Bishwa Chandra Adhikari, Bhotkar Ketan, Ravindra Patil, Eun Ha Choi, Kyu Chang Park

**Affiliations:** 1 Department of Information Display, Kyung Hee University, Dongdaemun-gu, Seoul, 02447 Republic of Korea; 2 Department of Electrical and Biological Physics, Plasma Bioscience Research Center (PBRC), Kwangwoon University, Seoul, 01897 Republic of Korea

**Keywords:** Applied optics, Applied physics

## Abstract

The focusing electrode plays an important role to reduce the electron beam trajectory with low dispersion and high brightness. This article summarizes the importance of the vertically aligned multi-walled carbon nanotube effect with the focusing electrode. First of all, the effect of electron beam trajectory is studied with the different heights, hole sizes, and applied voltage of the focusing electrode by the opera 3D simulation. The field emission electron beam spot is captured in the microchannel plate which helps to reduce the signal noise effect and damage of CNT tips by the joule heating effect. The high-dense bright spot is optimized at the focusing electrode hole size of 2 mm, and the height of 1 mm from the gate mesh electrode at the low bias voltage of − 200 V without the loss of current. The FWHM of the electron beam is calculated 0.9 mm with its opening angle of 0.9° which could be applicable in high-resolution multi-electron beam microscopy and nano-focused X-ray system technology.

## Introduction

The unique structure of carbon nanotubes (CNTs) has driven a new horizon of nanomaterial applications with their excellent mechanical properties^1^, chemical properties^2^, and electrical properties^3^, which lead to many potential applications. Field emission property is the excellent property among them due to the high aspect ratio with low atomic number^4,5^. The vertically aligned CNTs are crucial for the field emission electron beam of low dispersion^6^, low energy spread^4^, and high resolution^7^. The vertically aligned CNTs have a very low turn-on electric field (1–3 V/µm)^1,8^ and high field emission current density (> 1 A/cm^2^)^9^, which have unique field emission performance. The vertically aligned CNT is investigated to increase the performance of uniform field emission and lifetime. The vertically aligned CNTs are considered as cold cathode field emitter which possesses the advantage of small spot size, fast switch on, room temperature working, and low power^10^. In the field emission process, a voltage is applied between the emitter and the conducting electrode with the annular aperture to create a high electric field at the apex. This applied electric field bends the vacuum level and thus narrows the energy barrier between the electrons in the tip and the vacuum, leading the electron tunneling. Electrons are emitted as soon as the electric field is applied and consequently, this process has a fast switch-on time^11^. Nowadays, researchers are working in cold cathode field emission materials due to many applications such as microwave power amplifiers space technology, extreme ultraviolet (EUV) and UVC light, crystallization of amorphous materials, electron microscopy, and X-ray source technology^12–17^. It has many advantages as compared to the forest-type CNT emitter. The forest-type CNT emitter is easy to pattern, less time-consuming, and inexpensive, but it has many disadvantages such as non-uniform height, poor field emission performance, difficulty to focus, and the high screening effect^18,19^. The fabrication of conical nanostructure vertically aligned CNT makes them ideal candidates for enhancing cold cathode electron emission efficiency.

The field emission electron beam has the divergence angle where the field emission cathode is applied to the electron gun. The focusing electrode helps to reduce the beam divergence as an electrostatic focusing lens^20^. The small divergence of the electron beam gives a lower energy spread of the field emission beams, which is strongly suggested for the high brightness with the reduction of the beam spot^21,22^. The idea of two gate electrodes was considered by the W. B. Hermannsfeldt^23,24^ and the divergence angle is reduced to 2°. However, the two-gate concept is further studied by other researchers^20,25^. The focusing electrode is usually grounded or negatively biased such that the electrode repels diverging electron beams to the central axis for their focusing to designated phosphor pixels^26,27^. For the reduction of the beam divergence, the focusing electrode is positioned between the gate electrode and the anode with the optimized hole size and distance and biased voltage^11,26,27^. Many researchers are optimizing the focusing electrode structure, height, and size according to their experimental conditions but they are not finding the symmetrically distributed electron beam spot by using the cold cathode without loss of current^20,21,27–29^.

In this study, we captured the symmetrically distributed high-dense bright spot of field emission microscopy (FEM) image with its minimum size of 0.9 mm, and the beam divergence angle is calculated to be 0.9°. The result of the high-dense bright spot is compared in Table 1 in which researchers found the minimum electron beam spot according to their optimized conditions. According to this Table 1, the minimum electron beam spot is found in this experiment without loss of emission current and noise effect. In this experiment, the vertically aligned CNT emitters are perfectly aligned in the center of the gate mesh electrode of the triode system to increase the transmittance of the electron beam. This is the first experiment in which focusing electrode is playing an important role to reduce the electron beam trajectory while increasing the brightness and current density in the MCP of the vertically aligned one-island CNT emitter without loss of the current. This provides individual control of the emission current and emittance electron beam. Furthermore, the focusing electrode is negatively biased such that the electrode repels diverging electron beams to the center axis. The vertically aligned CNT is easy to fabricate and cheap as compared to the metallic tips. The high-dense bright spot of an electron beam is symmetrically distributed with uniform field emission on the phosphor screen of the microchannel plate (MCP) which may play an important role in multi-electron beam microscopy, and nano-focused X-ray technology.

**Table 1:** Comparison of high-dense bright spot of the electron beam in the anode.

S. N.	Source (Thermionic emission)	Source (Field emission)	Applied voltage (V)	Gate electrode (V)	Focusing electrode (V)	Emission current (µA)	Beam spot (mm)	Transmittance (%)	References
1	–	Laser induced metallic field emission array	0	91.0	–	100	2.50	–	^30^
2	–	Double gate field emitter (molybdenum)	− 80.0	0.00	− 69	0.4 × 10^–3^	1.34	87.0	^31^
3	–	Metallic tip field emitter (molybdenum)	− 72.0	0.00	− 69	1.9	2.00	85.0	^28^
4	–	Double gate field emitter (FEA1) (molybdenum)	− 80.0	0.00	− 69	0.7 × 10^–3^	1.02	90.0	^29^
5	–	Double gate field emitter (FEA2) (molybdenum)	− 128	0.00	− 69	0.7 × 10^–3^	2.17	80.0	^29^
6	–	Double gate field emitter (pulse diode gun) (molybdenum)	− 20.0 × 10^3^	54.0	− 54.0	–	0.62	–	^21^
7	–	Hairpin type field emission (W filament)	− 20.0 × 10^3^	− 17.4 × 10^3^	–	100	1.00	–	^32^
8	–	VACNT (Field emission)	− 900	0.00	–	0.34	2.71	90.0	^33^
9	–	VACNT (Field emission)	− 900	0.00	− 200	0.30	0.90	89.0	This result
10	Thermionic electron gun (W filament)	–	− 4.0 × 10^3^	− 600	–	25 × 10^3^	2.00	–	^34^

## Materials and methods

### Fabrication process of the carbon nanotube emitter

**Figure 1:**
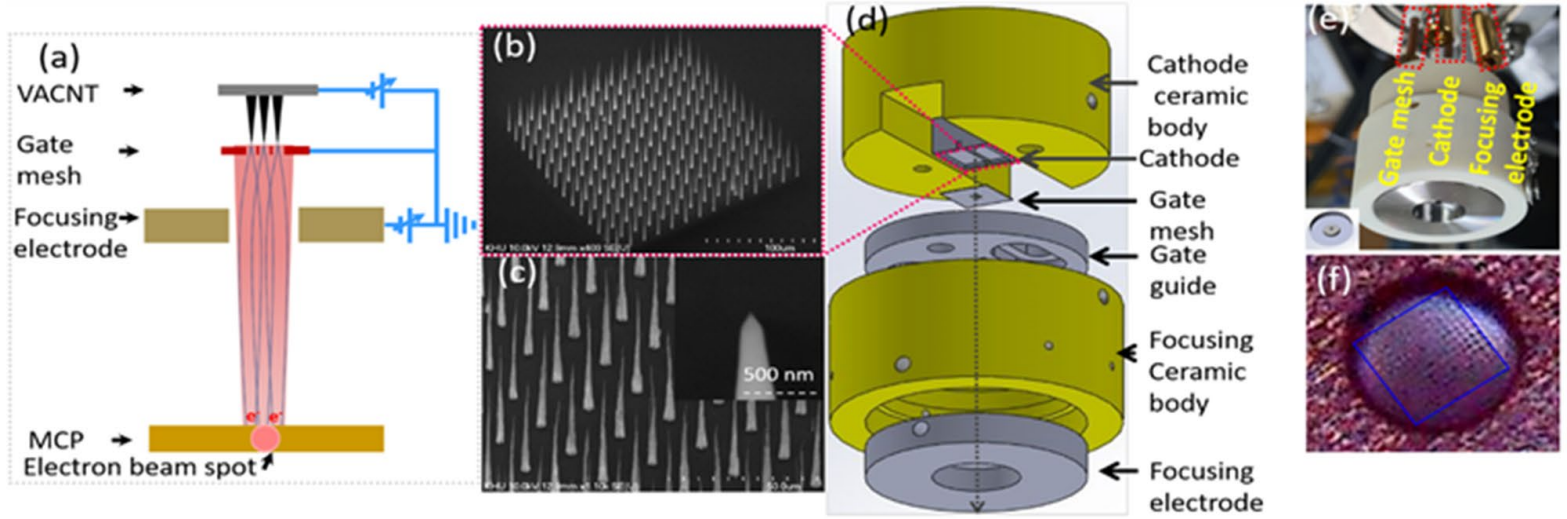
Image capturing process of the electron beam spot. (**a**) represents the schematic diagram of the power supply and the image capturing process of the field emission electron beam of 14 × 14 CNT emitters in the MCP. (**b**), and (**c**) represents the SEM image of 14 × 14 CNT emitters. Inset: (**c**) represents the single vertically aligned CNT emitter with its nanotip size of 50 nm. (**d**) represents the module design and beam alignment in solid work. (**e**) represents the compact CNT module with focusing electrode fixed inside the vacuum chamber. (**f**) represents the magnified picture of the CNT emitter alignment in the center of gate mesh and focusing electrode.

The CNT emitters are grown on the n-type silicon wafer substrate in the specified region, followed by the 30 nm nickel (Ni) deposition by radio frequency magnetron sputtering. The mask and dot pattern are designed with the dot diameter and pitch distance of 3 µm and 15 µm for the photolithography process^35^, respectively. A triode configuration of the direct current plasma enhanced chemical vapor deposition (DC-PECVD)^36^ is used to grow the CNT in the specified region with the bias cathode and mesh electrode voltage of − 600 V and + 300 V respectively. The C_2_H_2_: NH_3_ (18:200 sccm) gas flow rate is used to grow the CNT in that specified region with its working pressure of 1.8 Torr and the device temperature of 850 °C, respectively. After DC-PECVD grew, the 48% concentration of hydrofluoric acid (HF) is used as the etchant to make the smooth height of CNT^33^. Figure 1a represents the schematic diagram of the image capturing process of the field emission electron beam of 14 × 14 CNT emitters in the MCP. The morphological structure of the 14 × 14 CNT emitter is studied in scanning electron microscopy (SEM) which is shown in Fig. 1b and c, respectively. The tip shapes are smooth and uniform in height as shown in Fig. 1b and c respectively. The apex of the CNT emitter plays an important role in field emission with its high aspect ratio. In addition, the apex diameter of these CNT varies from 50 to 100 nm which depends upon their growth condition. Our CNT emitters have unique field emission properties with their high aspect ratio^7^. In our previous experiment^37^, we followed different dot sizes with different conditions of DC-PECVD such as called group 1, group 2, and group 3. The morphology structure is studied with the SEM and the structure is varying with its dot and tip size, respectively. The tip diameter, turn-on voltage, and beam size were analyzed in many samples. The group 1 sample is optimized^37^ which has a small tip diameter (50 nm) with a turn-on voltage of 950 V having high brightness and high stability of the field emission electron beam. Figure 1d represents the alignment design of the C-beam module in the solid work. The gate mesh electrode (SUS 304) is fixed in the gate guide and grounded. The height and hole diameter of the gate mesh is fixed at 250 µm from the cathode, and 300 µm, respectively. The different hole size of the focusing electrode is used at a distance of 1 mm from the gate mesh electrode. The ceramic body is used to fix the focusing electrode. Figure 1d shows the experimental connection of the C-beam module in the vacuum system for the measurement of FEM image. Figure 1e represents the C-beam module fixing inside the vacuum chamber. Figure 1f represents the alignment of the CNT emitters in the center of the focusing electrode.

### Microchannel plate

**Figure 2:**
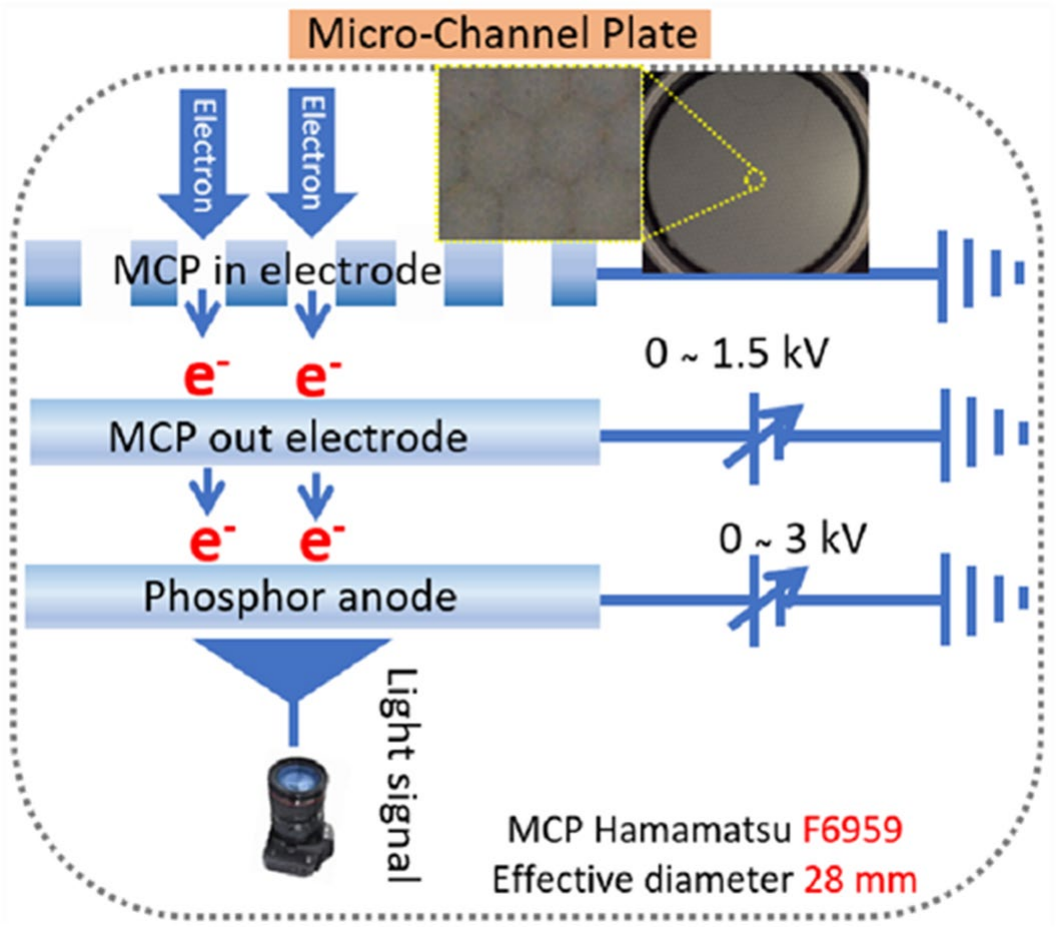
Schematic diagram of image capturing process in the MCP. (Inset: MCP image with its magnification).

The MCP is a compact electron multiplier of high gain^38,39^. It has been used in a wide range of particle and photon detection applications, such as ions, electrons, neutrons, X-rays, and UV rays^40–42^. It consists of a plate (disc) of an electrically insulating material (typically glass) and contains many tiny holes with a hexagonal array. The conventional phosphor screen has many defects with insufficient information on the electron beam trajectory which is affected by the signal noise due to the applied high voltage^43^. Furthermore, the sharp emitter can be damaged easily by a high electric field due to the joule heat effect^43,44^. The advantage of the MCP is a long life time^45^, high electronic gain^46^, fast response^47^, and very less signal noise^48^, respectively. The negative power supplied in the CNT emitters to generate the primary electron beam is called an incident electron beam. The primary electron beam is amplified near the threshold voltage by the generation of the secondary electron and protects the irreversible damage of the CNT during the vacuum arc. The entered primary electron beam strikes the channel of MCP and produces the secondary electron beam. This secondary electron accelerates toward the phosphor screen by an electric field developed by the voltage applied across both ends of the MCP. In the phosphor screen, the kinetic energy of the electrons is released as photons. The visible light can be captured in the phosphor screen of the MCP by using a digital single-lens reflex (DSLR) camera. Figure 2 shows the schematic diagram of the MCP with a three-electrode system called MCP- in, MCP-out, and phosphor anode, respectively. The inset of Fig. 2 shows the hexagonal structured tiny hole of the MCP. The MCP (Hamamatsu MCP F6959, Japan) is fixed at a distance of 25 mm from the cathode to measure the FEM image on the phosphor screen. The setup and power supply detail of the MCP is explained in our previous experiment^33^.

**Figure 3:**
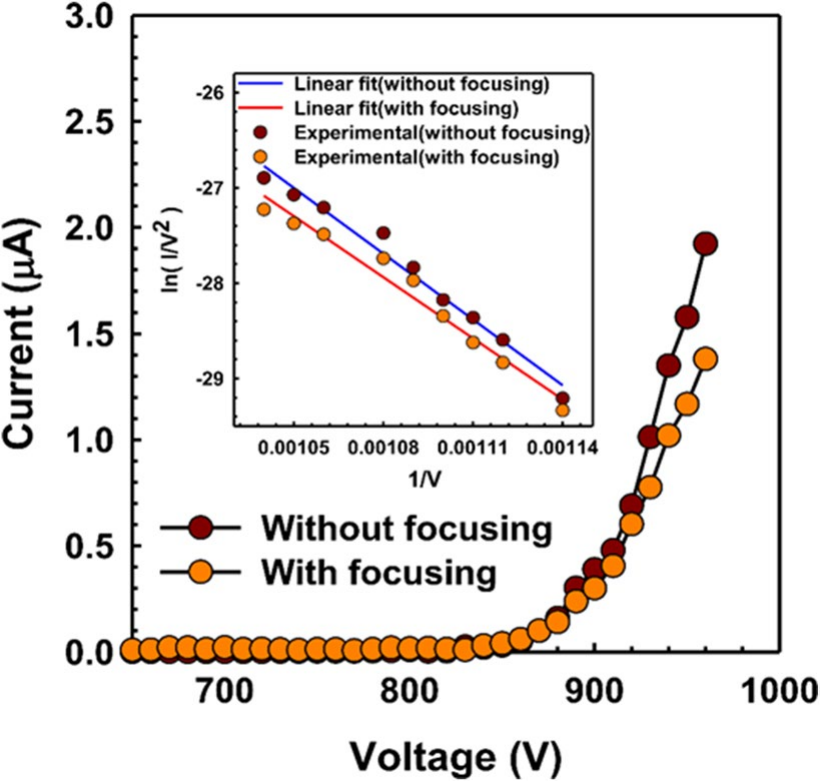
I–V characteristics of the electron beams at the anode without focusing and with focusing electrode at − 200 V. Inset: F-N plot with and without focusing electrode of 14 × 14 CNT emitters.

## Results and discussion

### I–V characteristics of the CNT emitters

The vertically aligned CNT emitters are atomically sharp tips that enhance the field emission properties^49^. The I–V characteristics play an important role to explain the uniform filed emission performance which is influenced by the types of materials, arrangements, and surface morphology of the emitters, respectively^44^. The properties of vertically aligned CNT emitters, namely a work function (ϕ), high aspect ratio, and excellent electrical properties are the important key which is optimized in our previous experiment^33,37^. Figure 3 exhibits the I–V characteristics of the one-island cone-shaped CNT with 14 × 14 emitters as the function of the applied voltage. In Fig. 3, when the applied voltage is increasing the emission current is also exponentially increasing after the threshold voltage of 810 V. In 900 V, the emission current is measured at 0.34 µA, and 0.30 µA without focusing and with a focusing electrode (− 200 V), respectively. After 900 V, the emission current is increasing dramatically with the applied voltage because of the high electric field effect between the gate electrode and the focusing electrode. The current density of the electron beam spot is increasing 8 times from 5.89 µA/cm^2^ to 47.15 µA/cm^2^ in the anode with the optimized condition of focusing electrode. This optimized focusing electrode is playing an important role to push the electrons toward the central axis and increasing the current density and brightness of the beam spot without loss of current.

### Field emission microscopy image with the focusing electrode

**Figure 4:**
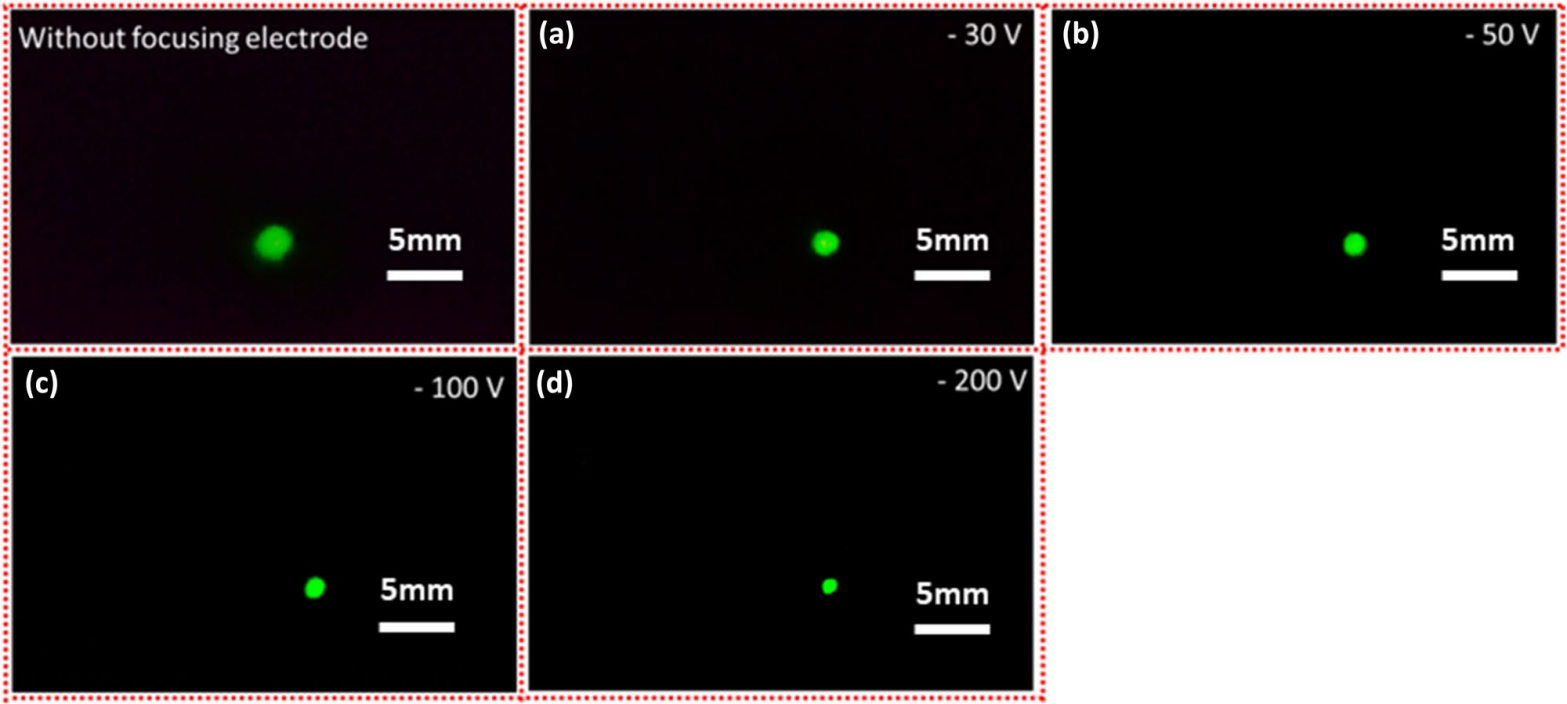
FEM image of the CNT emitter in the MCP without focusing electrode and with the focusing electrode hole size of 2 mm under focusing bias of − 30, − 50, − 100, − 150, and − 200 V, respectively.

FEM image provides quantitative information about the microscopic properties of the CNT emitters. A focusing electrode is used to evaluate the electron beam spot in the MCP. The FEM image is captured in the center of the MCP, which provides the real electron beam trajectory. The incident electron beam passes towards the MCP-in electrode with its beam axis. When electrons depart towards the phosphor screen of MCP, it is converted into photons and green light emits. Figure 4 exhibits the FEM image of the 14 × 14 CNT emitters in the center of the MCP in which the applied voltage and the brightness shutter time were fixed at 900 V, and 1/3 s, respectively during the whole experiment, according to our previous experiment^33^. In our previous experiment, the FEM image-capturing process was optimized by reducing the signal–noise ratio without focusing electrode with its real beam spot size of 2.71 mm. The potential difference between the CNT emitter and the gate mesh is fixed at 900 V. The applied voltage in MCP-out electrode and the phosphor electrode is fixed at 375 V and 1600 V, respectively in this experiment. The electron beam spot size of the 14 × 14 CNT emitters can be analyzed with the help of the FEM image. The symmetrically distributed electron beam spot is well described by the Gaussian distribution^7,50^, $G(x) = G(0) + A.\exp \left\{ {\left. { - 0.5\left( {\frac{{x - x_{0} }}{\sigma }} \right)^{2} } \right\}} \right.$, Where $A,x_{0}$, and $\sigma$ represents the peak intensity, mean (maximum peak intensity), and standard deviation of the distribution respectively. In the electron beam spot profile, the width of the intermediate of the maximum intensity value represents the FWHM which is expressed as $2\sqrt {2\ln } \sigma$. In Fig. 4a–d, the focusing electrode voltage is − 30 V, − 50 V, − 100 V, − 150 V, and − 200 V in which the FWHM of FEM electron beam spot is calculated to be 2.23 mm, 2.00 mm, 1.78 mm, 1.61 mm, 0.90 mm, respectively in which the electron beam spot without focusing electrode is calculated to be 2.71 mm. The size of high-dense bright spot is continuously reducing and the brightness is increasing with increasing the current density at the maximum collimation due to the reduction of the opening angle of the electron beams^28,29,31^. In the focusing lens, while the electrons enter in the strong electric field, these electrons come closer to the central axis due to the repulsive force of the focuser in the optimized conditions. Furthermore, when the focusing bias of focuser is increasing, these electrons cannot enter to the focuser due to high repulsive force. The cathode bias is fixed at the 900 V and the focusing bias is changing from − 30 V to − 200 V. However, the power is less than 100 mW in the focusing bais of − 200 V.

**Figure 5:**
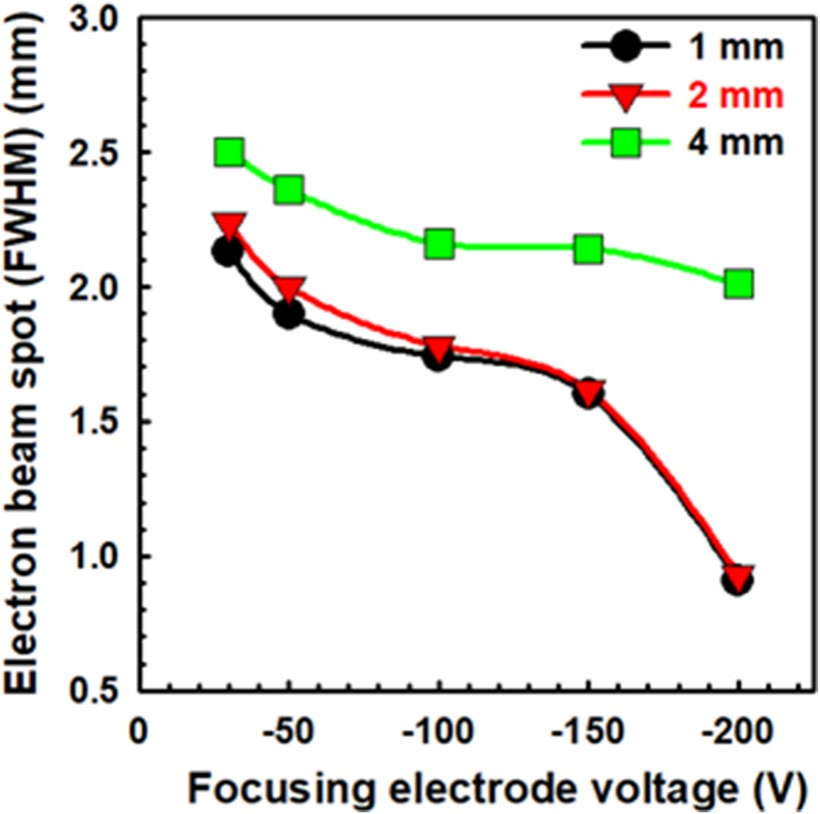
High-dense bright spot of the electron beam with the different focusing electrode hole size.

**Figure 6:**
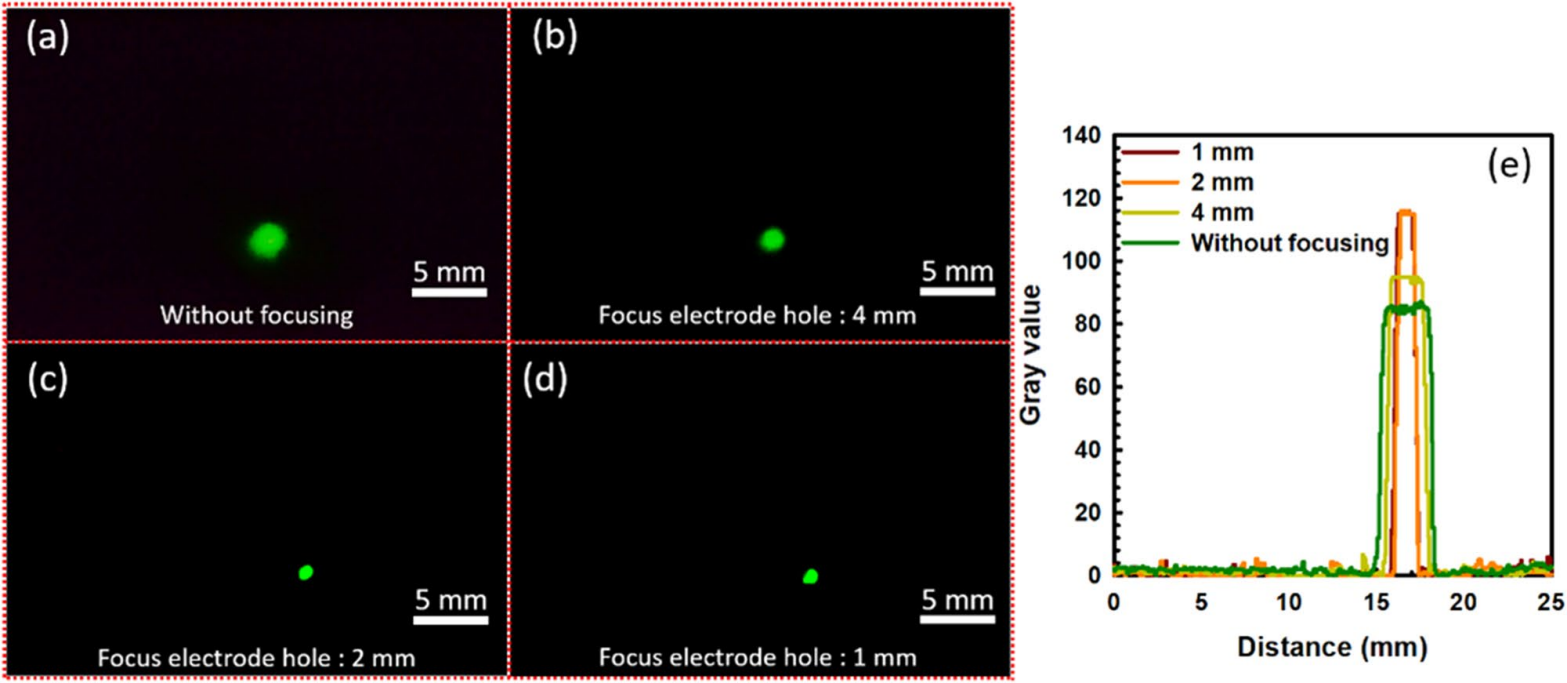
(**a**–**d**) is the high-dense electron beam spot in the MCP without focusing, with focusing electrode hole size of 4 mm, 2 mm and 1 mm, at focusing electrode voltage of − 200 V, respectively. (**e**) represents the electron beam intensity with different focusing electrode hole size at − 200 V, respectively.

Figure 5 represents the comparison of the high-dense bright spot of the field emission electron beam with different focusing electrode holes such as 1 mm, 2 mm, 4 mm, respectively. The cathode voltage is fixed at − 900 V and the focusing electrode voltage is continuously changing from − 30 V to − 200 V in which the electron beam spot is reducing. The electron beam spot without the focusing electrode is calculated to be 2.71 mm. Under the focusing electrode voltage of − 200 V, the FWHM of high-dense bright spot is calculated to be 0.89 mm and 0.90 mm in the focusing electrode hole of 1 mm, and 2 mm, respectively. Experimentally, it is found that the electron beam spot is similar at − 200 V between the focusing electrode hole of 1 mm and 2 mm. Figure 6a–d represents the electron beam spot without focusing electrode, with focusing electrode hole size of 4 mm, 2 mm, and 1 mm at focusing electrode voltage of − 200 V, respectively. The intensity of the electron beam spot is continuously increasing and the beam spot is continuously decreasing with the focusing bias, as shown in Fig. 6e. The beam spot and intensity of the electron beam spot are found similar to each other at the 1 mm and 2 mm of the focusing electrode hole. Experimentally, it is confirmed that the focusing electrode hole size of 2 mm is optimized for the vertically aligned CNT emitters to reduce the high-dense bright spot with high current charge density and brightness. The fabrication of 2 mm hole focusing electrode is easier and cheaper as compared to the 1 mm hole size of the focusing electrode. Figures 4 and 6 confirm that the high-dense bright spot of the electron beam is symmetrically distributed in the phosphor screen with the uniform field emission performance from the cone-shaped vertically aligned 14 × 14 CNT emitters. The diameter of the high-dense electron beam spot is reduced 3 times from the original position under the focusing electrode voltage of − 200 V. In this experiment, the experimental result is compared with the simulation result in detail which confirm the electron beam trajectory of the CNT emitters.

**Figure 7:**
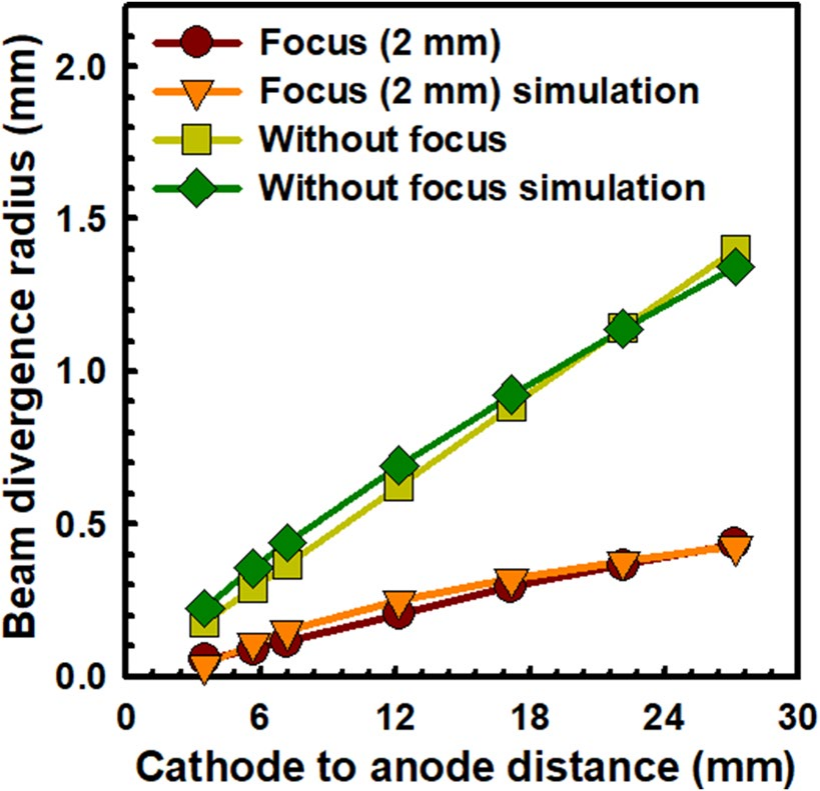
Comparison the simulation results with experimental results without and with focusing electrode (hole size of 2 mm) in the variation of the gap distance between cathode source to the phosphor electrode.

**Figure 8:**
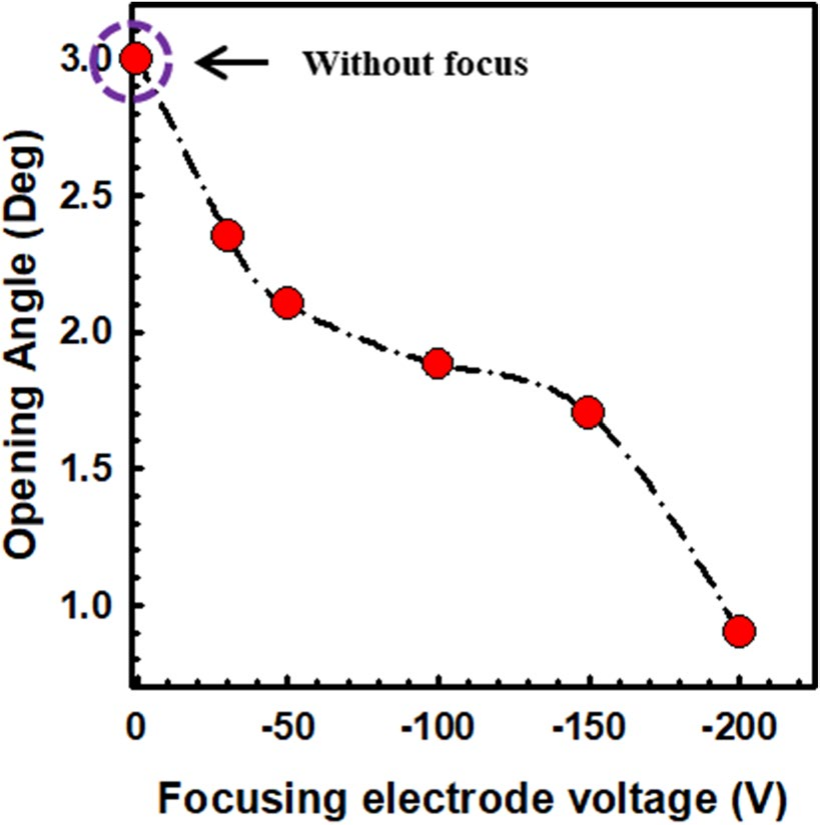
Variation of opening angle of the electron beam trajectory with focusing electrode (hole size of 2 mm) under the variation of the focusing electrode voltage.

Figure 7 explains the variation of the beam divergence without and with the focusing electrode (hole size of 2 mm) at − 200 V. According to our previous experiment^33,37^, the electron beam trajectory follows the curve fitting parameters for the simulation with the variation of the cathode height from the phosphor screen. From Fig. 7, it is clear that the simulation result is consistent with the experimental results with the reduction of the anode distance. The divergence of the electron beam depends upon the size of the electron beams, cathode to anode distance, size of the focusing electrode, and gap distance of the focusing electrode from the cathode^51,52^. Figures 4, 5, and 6 confirm that the electron beam is symmetrically distributed with uniform field emission to the MCP. The reduction opening angle of the electron beam is defined as^29,31^, $$D/L = 2\tan \theta /1 + \left( {\sqrt {1 + {\raise0.7ex\hbox{${V_{an} }$} \!\mathord{\left/ {\vphantom {{V_{an} } {\left| {V_{em} } \right|{\text{Cos}}^{2} \theta }}}\right.\kern-0pt} \!\lower0.7ex\hbox{${\left| {V_{em} } \right|{\text{Cos}}^{2} \theta }$}}} } \right)$$ where, D, L, V_an_, V_em_ is the half-width of the half-maximum intensity of the electron beam spot on the phosphor screen, distance from the point source to the phosphor (27.17 mm), applied voltage to the MCP-in electrode ( 0 V), and applied voltage to the CNT emitters (900 V), respectively. Figure 8 exhibits the reduction of the opening angle of the electron beam trajectory from 3° to 0.9° in the focusing electrode hole size of 2 mm. The opening angle of the high-dense electron beam spot reduces approximately 3 times from its initial position, which plays an important role in the low dispersion of electron beams with high current density. The minimum angle is calculated at the focusing electrode of − 200 V.

### Beam spot analysis with simulation

**Figure 9:**
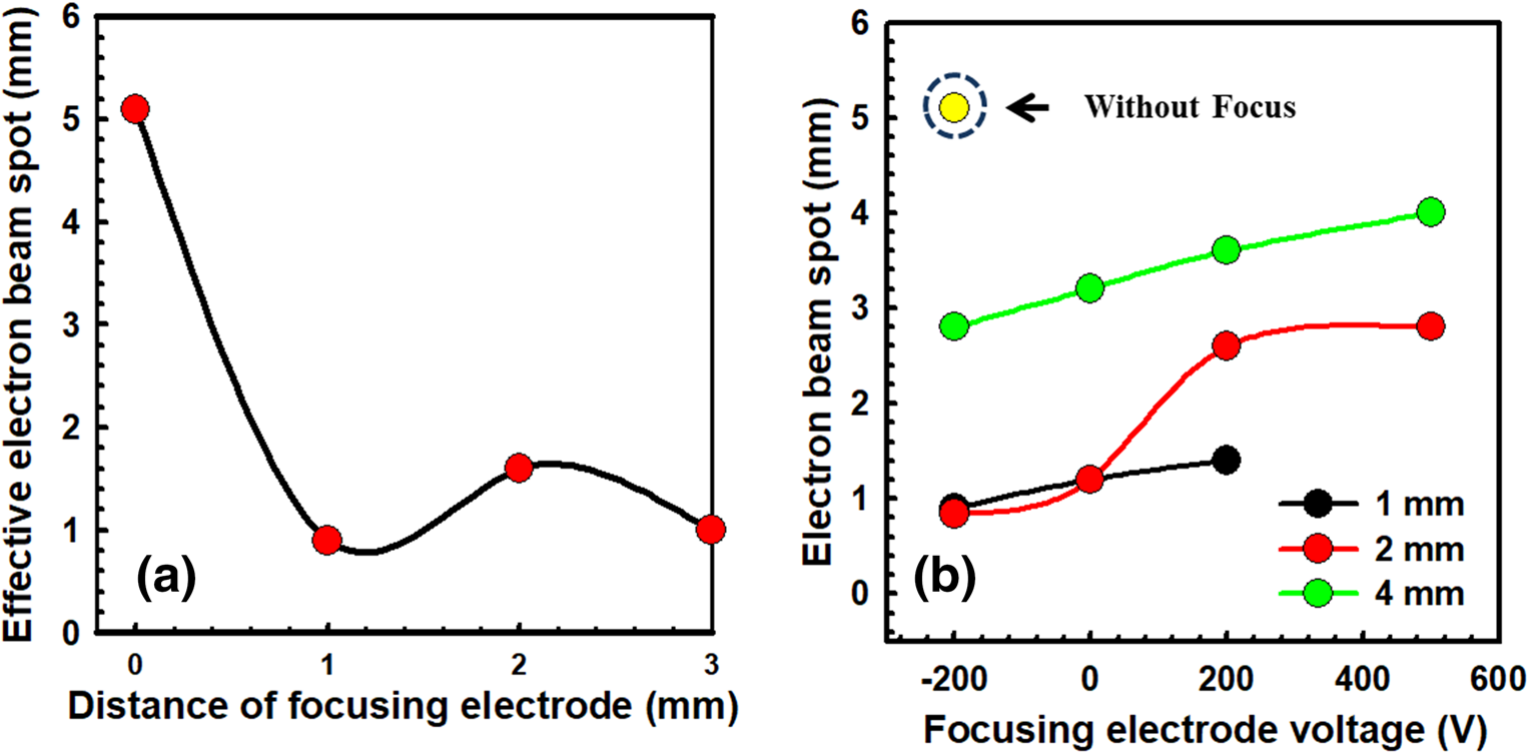
Opera 3D simulation of beam divergence of the electron beam spot. (**a**) Electron beam spot with various focusing electrode height from gate mesh. (**b**) Electron beam spot with various applied voltage with the different focusing electrode hole.

Beam simulation results have been valuable for designing the beam module^34^ and understanding the beam trajectory^53^ as well as applied voltage^54^. The computer simulation helps to optimize and construct the electron beam configurations in the diode and triode systems. 3D opera simulation is considered to the accelerator design as follows: hole diameter, applied voltage and height of the focusing electrode from the gate mesh. In this experiment, the 3D opera simulation parameters are explained in detail in the Table 2. Due to the high electric field effect of focusing electrode, the emitted electrons from the gate mesh converge to the central axis which reduces the electron beam spot and increases the beam intensity in the anode. Figure 9a exhibits opera 3D simulation results of bright spot of field emission electron beam with the variation of height of focusing electrode from the gate mesh electrode. To opmized the height of focusing electrode, hole size of focusing electrode is fixed at a diameter of 2 mm. The minimum electron beam spot is found at 1 mm height of focusing electrode from the gate mesh electrode. After 1 mm height of focusing electrode, beam spot is increasing because the emitted electrons from the focusing electrode is diverging. Figure 9b represents the high dense electron beam spot in which focusing electrode hole size is varied from 1 to 4 mm as well as the applied voltage is varied from − 200 V to 500 V, respectively. The minimum electron beam spot is found in 2 mm hole size of the focusing electrode. The minimum electron beam spot of 0.84 mm is calculated in the focusing electrode hole size of 2 mm at the applied voltage of − 200 V because the diverged electrons are highly focused at the center of the beam axis.

**Table 2:** 3D opera simulation parameters.

Simulation parameters	
Emitter type	Fowler–Nordheim field emission
Cathode voltage	Grounded
Gate voltage	1 kV
Anode voltage	5 kV
Emitter temperature	400 K
Emitter work function	4.5 eV
Particle charge	− 1
Field enhancement factor	1
Distance between cathode &gate	150 µm
Distance between gate & focuser	1 mm
Gate mesh hole size	0.4 mm
Focusing electrode hole size	1 mm, 2 mm and 4 mm
Electron energy	5 keV (Anode bias of 5 kV)

**Figure 10:**
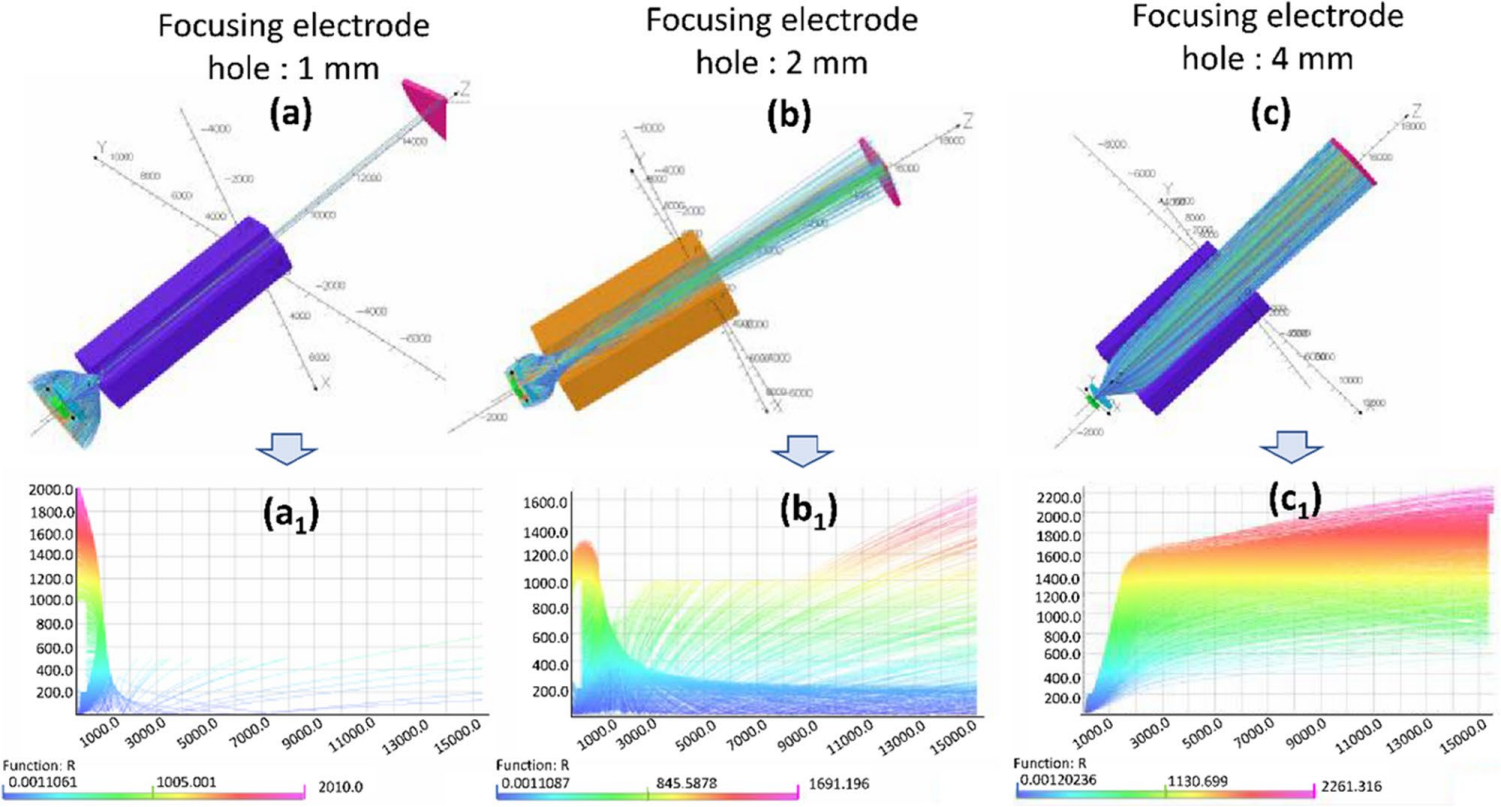
Opera 3D simulation results of the field emission electron beam with the variation of the focusing electrode hole size at focusing bias − 200 V.

**Figure 11:**
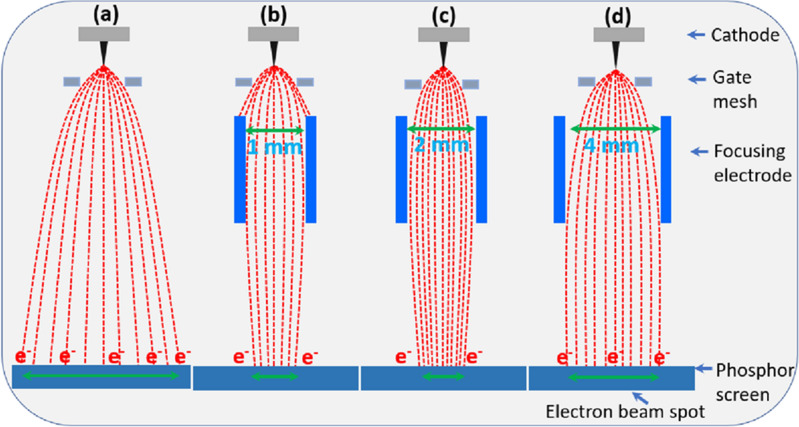
Schematic diagram of beam divergence of the C-beam with various electrode hole size at − 200 V focusing bias condition.

Figure 10 explain the opera 3D simulation of electron beam trajectory and effective radius with the different focusing electrode hole size. In Fig. 10a,b and c, the minimum electron beam spot is calculated to be 0.9 mm, 0.84 mm and 3 mm with different hole size of 1 mm, 2 mm, and 4 mm, respectively at − 200 V. In case of focusing lens hole size of 1 mm and 4 mm, the electron beam spot is repelled from the gate mesh, and unable to focus highly so that the electron beam spot is affected. Figure 11 explains the schematic diagram for the understanding of the electron beam trajectory of the CNT emitters (one-island source). The CNT emitter is fabricated in the specified region with its height of 40 µm. The gate mesh controls the extracted electron beams and helps to pass towards anode. The focusing electrode helps to focus the diverged electrons towards the central axis. The optimization parameters of the beam are very important to focus electrons at the central axis. Figure 11a shows electron beam trajectory without focusing electrode in which electron beams diverge with the distance between gate and anode. Figure 11b shows the small hole size of 1 mm of the focusing electrode, in which electrons are diverged due to the electric field effect and passing away from the central axis. Figure 11d exhibits the 4 mm hole size of the of focusing electrode in which electrons are unable to focus highly at the central axis. Furthermore, Fig. 11c shows the 2 mm of optimized focusing electrode hole size in which electrons are passing towards the central axis with minimum beam spots. So that, the electron beam size is optimized by the opera 3D simulation and compared with the experimental results in the MCP. The minimum electron beam spot is obtained at 2 mm of focusing electrode hole by the simulation results as well as the experimental results.

## Conclusion

The vertically aligned cone-shaped CNTs with 14 × 14 emitters were fabricated as a single electron beam source in the Si wafer by the sputtering, photolithography, and PE-CVD process. To obtain a smaller beam spot size and lower angular dispersion with high current density, the carbon nanotube-based cold cathode electron beam (C-beam) was fabricated and simulated with various focusing schemes. The opera 3D simulation helped to understand and confirm the height from gate mesh, hole size and bias voltage of the focusing electrode. The 14 × 14 CNT emitter is one-island called the single source. The beam source is perfectly aligned at the center of the gate mesh, focusing electrode, and phosphor of the MCP, respectively. The minimum electron beam spot with high brightness is successfully demonstrated and optimized by the focusing electrode with a small opening angle of 0.9° by the reduction of the noise effect in the MCP. The high-dense bright spot is optimized at 2 mm of the focusing electrode hole size with its height of 1 mm from the gate mesh electrode with the low bias voltage of − 200 V without loss of current. The minimum electron beam spot of the CNT emitters is calculated 0.9 mm in the MCP and 8 times enhanced beam current density compare to the non-focused scheme, which could be applicable in the high-resolution multi-electron beam microscopy, and nano-focused X-ray system.

## Data Availability

The authors declare that most data supporting the findings of this study are available within the paper file. The rest of the data generated during and/or analyzed during the current study are available from the corresponding author upon reasonable request.
